# Indents

**Published:** 1901-02-15

**Authors:** 


### INDENTS.

**☞ Brief Articles of Technical or Scientific Value will receive notice and comment. Contributions invited.**

SCALING SENSITIVE TEETH.—Prepare equal parts of iodid of potassium and of iodin crystals, in a saturated aqueous solution. Also a saturated aqueous solution of sulphate of zinc. Mix together equal parts of the supernatant fluid from the solutions and apply to the sensitive surfaces—E. A. Bogue, *Items of Interest*.

TREATMENT OF CANAL AFTER PULP REMOVAL.—If infection at the apex is suspected, treat with sulphuric acid and sodium dioxid, and seal in the tooth for a few days oil of cloves containing 1 percent of formaldehyd. If there is no soreness at the next sitting, proceed to fill.—Harold Clark, *Dominion Dental Journal*.

ARREST OF HEMORRHAGE AFTER TOOTH EXTRACTION.—With hypodermic syringe inject a few drops of a 3 percent solution of pyrozone, or peroxid of hydrogen, in the apex of each alveolar socket and around the gum margin. The instantaneous expansion stops all hemorrhage immediately.—W. M. Barnett, *Dental Digest*.

THE COMPOSITION AND USE OF TOOTH POWDER.—Medicated dentifrices are not specially indicated in ordinarily healthy mouths. Their function is simply cleansing the teeth. The question as to whether they be acid or alkaline in reaction is of no considerable importance, unless it interfere with their detergent values or injure the tissues. Agreeable concoctions lead to more persistent use. It is infinitely more important that people be taught to use the teeth as much as possible in masticating their food.—Dr. G. V. I. Black, *Dental Cosmos.*

Making Porcelain Inlays.—"Gumption" is of as much value as anything else. Make the inlay thick enough for strength and cement attachment. Save as much of the lingual surface of incisors as possible to assist in retention of the inlay and prevent the wash of the tongue on the cement. Where their labial enamel is left, be careful to use a cement the same color as the tooth. Always get free access to proximal cavities. In enameling the inlay biscuit, when several shades are required, they will blend if laid on moist and in contact. In devitalized teeth, color darker than the tooth; in light colored teeth, shade lighter if anything. Wash tooth cavity with alcohol previous to inserting the inlay. Harmonize the cement color with the tooth and inlay.—F. J. Capon, *Dental Cosmos.*

To Control Hemorrhage in Hemophilic Patients.—After extraction pack the alveoli with iodoform gauze and cover it with a ball of cotton, to be retained in position by rubber dam attached with strong ligatures to adjoining teeth. This makes gentle pressure on the wound and holds the gauze in place, and by cutting away excessive rubber dam it may work with comfort several hours or days and does not interfere with eating or irrigation of the mouth.—Johann Franck, *Dental Cosmos.*

The Vacuum Chamber.—Use a small rather than a large chamber, medium thickness, nearly as thick as a nickle five cent piece. Do not cover the chamber in impression or build it on the model. Study the mouth, noting variations, and make and adopt the chamber to each case as required.—R. H. Nones, *Items of Interest*.

Sodium Peroxide as a Disinfectant.—It may be used in a saturated solution, but as the oxygen is so easily disassociated in this form it may be used in the powder form, carrying it into the pulp canal on a broach, when the moisture of the tooth tissues sets up a rapid effervescence, which may be intensified by the addition of a 10 percent solution of sulphuric or hydrochloric acid. This treatment effectively disinfects putrid pulp canals and bleaches the tooth so that unsightly contrasts are seldom produced.—C. J. Peters, January *Items of Interest*.

Nirvanin as a Dental Anesthetic.—Hypodermic injections are profound and of long duration; extractions are, with few exceptions, painless; produces no œdema or sloughing. Solutions should not exceed 5 percent. The length of time topical applications are continued has much to do with their effectiveness. It is claimed that a 10 percent solution will obtund sensitive dentin, but I have had no success with it used for this purpose.—R. C. Brewster, January *Items of Interest*.

Chloretone Anesthetic in Pyorrhea.—Spray the pockets first with a strained solution of suprarenal capsule to drive the excessive blood out of the tissues; then apply an ether solution of chloretone. This will enable more thorough scaling of the teeth, with little or no pain.—Dr. Ferris, *Items of Interest*.

Practicability of Porcelain Inlays.—While somewhat limited in application, the porcelain inlay occupies a high and indisputable sphere of usefulness and has come to stay. Up-to-date dentists must know how to make them and where to place them. Enthusiasts have been too extravagant, leading their imitators into disastrous failures. But where indicated and properly adjusted they are useful and artistic in the highest degree.—H. J. Goslee, *Dental Digest*.

GUTTA PERCHA ROOT FILLINGS.—I believe gutta percha to be the best root filling if the root canal is absolutely dry; if it is not dry, then it is one of the worst materials that can be used. Dry the root canal with iodoform dissolved in chloroform or ether, followed by a heated point or hot air. Use a gutta percha point slightly smaller than the canal, and before introducing it moisten with chloroform and place a bit of iodoform on the end of the point. Then force the point to the extreme apex of the root; again use the hot air and soften the gutta percha to a creamy consistency, and with pressure force it into every part of the canal. In this way I believe the canal will be as well closed as is practicable by any method.—T. W. Onderdonk, January *International Journal*.

EUCALYPTO-PERCHA.—Owing to my belief that chlora-percha occupies less space in root canals after the chloroform has evaporated, I have been using for seven years a solution of gutta percha in eucalyptol, as there is little or no contraction, and any excess of oil penetrates the tubuli and acts as a durable antiseptic.—Dr. Leroy, January *International Journal*.

